# *Agrobacterium* VirE3 Uses Its Two Tandem Domains at the C-Terminus to Retain Its Companion VirE2 on the Cytoplasmic Side of the Host Plasma Membrane

**DOI:** 10.3389/fpls.2020.00464

**Published:** 2020-04-21

**Authors:** Xiaoyang Li, Tingting Zhu, Haitao Tu, Shen Q. Pan

**Affiliations:** ^1^Department of Biological Sciences, National University of Singapore, Singapore, Singapore; ^2^School of Stomatology and Medicine, Foshan Institute of Molecular Bio-Engineering, Foshan University, Foshan, China

**Keywords:** *Agrobacterium*, VirE3, VirE2, T-complex, T-DNA, plasma membrane

## Abstract

*Agrobacterium tumefaciens* is the causal agent of crown gall disease in nature; in the laboratory the bacterium is widely used for plant genetic modification. The bacterium delivers a single-stranded transferred DNA (T-DNA) and a group of crucial virulence proteins into host cells. A putative T-complex is formed inside host cells that is composed of T-DNA and virulence proteins VirD2 and VirE2, which protect the foreign DNA from degradation and guide its way into the host nucleus. However, little is known about how the T-complex is assembled inside host cells. We combined the split-GFP and split-sfCherry labeling systems to study the interaction of *Agrobacterium*-delivered VirE2 and VirE3 in host cells. Our results indicated that VirE2 co-localized with VirE3 on the cytoplasmic side of the host cellular membrane upon the delivery. We identified and characterized two tandem domains at the VirE3 C-terminus that interacted with VirE2 *in vitro*. Deletion of these two domains abolished the VirE2 accumulation on the host plasma membrane and affected the transformation. Furthermore, the two VirE2-interacting domains of VirE3 exhibited different affinities with VirE2. Collectively, this study demonstrates that the anchorage protein VirE3 uses the two tandem VirE2-interacting domains to facilitate VirE2 protection for T-DNA at the cytoplasmic side of the host cell entrance.

## Introduction

*Agrobacterium tumefaciens* is a soil-borne phytopathogen that causes crown gall disease in a variety of plant species in nature (Nester, 2014). This bacterium is well-known for its unique ability for inter–kingdom DNA transfer (Chilton et al., 1977; Zambryski et al., 1980; Albright et al., 1987) and has been widely used in plant biotechnology for decades (Tzfira and Citovsky, 2006; Hwang et al., 2017). Under laboratory conditions, *A. tumefaciens* is also able to transfer DNA into yeast (Bundock et al., 1995; Piers et al., 1996), algae (Kathiresan et al., 2009) and fungal cells (de Groot et al., 1998); it is widely used as a genetic vector for different cells (Michielse et al., 2005; Tzfira and Citovsky, 2006; Idnurm et al., 2017).

*Agrobacterium*-mediated transformation (AMT) is a multistep process facilitated by a series of virulence proteins encoded by *vir* genes on the Ti plasmid (Stachel and Nester, 1986; McCullen and Binns, 2006; Pitzschke and Hirt, 2010). In addition, various host proteins are also shown to be involved in this transformation process (Citovsky et al., 2007; Gelvin, 2010). Initially, the bacterium senses the plant phenolic and monosaccharide inducers, which activates the two-component VirA/VirG system; this subsequently results in rapid expression of all the *vir* genes (Stachel et al., 1985; Stachel and Zambryski, 1986; Shimoda et al., 1990). Virulence proteins VirD1 and VirD2 form a nuclease complex and generate a single-stranded (ss) transferred DNA (T-DNA) from the Ti plasmid (Wang et al., 1984; Yanofsky et al., 1986; Scheiffele et al., 1995); the T-DNA remains covalently attached to VirD2 and is transferred into the host cell through the VirB/D4 type IV secretion system (T4SS) (Cascales and Christie, 2003, 2004). The T4SS is a membrane-spanning transporter complex composed of twelve virulence proteins including VirB1-11 and VirD4 (Christie et al., 2005; Chandran Darbari and Waksman, 2015). VirB1-11 are the main structure components of this membrane-associated export apparatus while VirD4 is a type IV coupling protein located at the entrance of the secretion channel, which mainly acts to deliver substrate proteins into the channel (Cabezon et al., 1997).

Besides T-DNA and VirD2, at least four other virulence proteins are delivered into the host cell through the T4SS, including VirD5, VirE2, VirE3, and VirF (Vergunst et al., 2000, 2005; Schrammeijer et al., 2003). Among these translocated effectors, VirE2 is capable of binding ssDNA without sequence specificity (Christie et al., 1988; Citovsky et al., 1989; Sen et al., 1989). Moreover, VirE2 could self-interact to form filamentous structure *in vitro* and *in vivo* in a head-to-tail manner (Frenkiel-Krispin et al., 2007; Dym et al., 2008; Li et al., 2014). Thus, it is hypothesized that VirE2 can coat the T-DNA to form the “T-complex” and protect it from nucleolytic degradation inside host cells (Yusibov et al., 1994; Rossi et al., 1996). Two putative nuclear localization signals (NLSs) on VirE2 have been reported and it was shown that VirE2 could directly interact with several plant importin-α isoforms (Bhattacharjee et al., 2008). Furthermore, VirE2 was also shown to target the host nucleus with the help of a plant protein VIP1 (Tzfira et al., 2001; Li et al., 2005). Thus, VirE2 may function together with VirD2 to facilitate nuclear import of the T-complex. Although the T-complex is supported by various genetic and *in vitro* studies, it is still not clear how the complex structure is formed inside host cells.

VirE3 is a virulence protein conserved in *Agrobacterium* and rhizobia species (Li et al., 2018). It was reported that VirE3 possessed two NLSs and could interact with *Arabidopsis thaliana* importin-α to facilitate nuclear targeting of VirE2 (Lacroix et al., 2005). Moreover, the transcriptional activity of VirE3 has also been reported, indicating its multiple roles during the transformation process (Garcia-Rodriguez et al., 2006; Niu et al., 2015).

Recently, we demonstrated that VirE3 was an anchorage protein, as it could target the host plasma membrane through a conserved membrane-localization domain at the host cell entry site; by directly interacting with VirE2, VirE3 could retain VirE2 temporarily on the cytoplasmic side of the host plasma membrane and thus facilitate the T-DNA coating and T-complex assembly (Li et al., 2018).

In this study, we combined the split-GFP and split-sfCherry systems to visualize both VirE2 and VirE3 upon their delivery into host cells. Our data indicated that VirE2 and VirE3 physically associated with each other at the host cell border. Moreover, two conserved VirE2-interacting domains were identified at VirE3 C-terminus; these two domains functioned cooperatively to retain VirE2 on the cytoplasmic side of the host plasma membrane through direct interactions, which may also facilitate VirE2 self-aggregation and T-DNA coating at the host cell entry site.

## Materials and Methods

### Strains, Plasmids, Primers and Growth Conditions

*A. tumefaciens* strains, plasmids and primers used in this study are listed in Supplementary Tables S1–S3, respectively. *A. tumefaciens* strains were grown at 28°C in Luria-Bertani (LB) medium supplemented with kanamycin (50 μg ml^−1^) as necessary. The yeast strain AH109 was cultured in YPDA medium.

### Plant Materials and Growth Conditions

*N. benthamiana* wild-type and transgenic line Nb308A (expressing GFP1–10 and DsRed) plants were grown at 22°C under a 16-h light/8-h dark photoperiod.

### *A. tumefaciens* Mutants Construction

*A. tumefaciens* mutants were generated using a *sacB*-based gene replacement strategy (Hoang et al., 1998).

#### EHA105virE2::sfCherry11 and EHA105-VirE2::sfCherry11-VirE3::GFP11

Flanking sequences of the *virE2* permissive site (Zhou and Christie, 1999) were amplified from the total DNA of *A. tumefaciens* strain EHA105 with primer pair E1001/E1002 or E1003/E1004. The PCR products were further amplified with primer pair E1005/E1006 using overlapping PCR, digested with XbaI and XhoI and inserted into pEx18Km to generate pEx18Km-VirE2::sfCherry11. pEx18Km-VirE2::sfCherry11 was then introduced into *A. tumefaciens* strain EHA105 or EHA105*virE3::GFP11* to generate EHA105*virE2::sfCherry11* or EHA105-VirE2::sfCherry11-VirE3::GFP11, respectively.

#### EHA105ΔVirE3(522-541) and Its Derivatives

To generate *A. tumefaciens* strains EHA105ΔVirE3(522-541) and EHA105*virE2::GFP11*ΔVirE3(522-541), the flanking sequences were amplified from the total DNA of *A. tumefaciens* strain EHA105 with primer pair E1007/E1008 or E1009/E1010. The PCR products were further amplified with primer pair E1011/E1012 using overlapping PCR, digested with XbaI and SalI and inserted into pEx18Km to generate pEx18Km-ΔVirE3(522-541). pEx18Km-ΔVirE3(522-541) was then introduced into *A. tumefaciens* strain EHA105 or EHA105*virE2::GFP11* to generate EHA105ΔVirE3(522-541) or EHA105*virE2::GFP11*ΔVirE3(522-541), respectively. To generate *A. tumefaciens* strain EHA105*virE3::GFP11*ΔVirE3(522-541), the flanking sequences were amplified from the total DNA of *A. tumefaciens* strain EHA105*virE3::GFP11* with primer pair E1007/E1008 or E1009/E1010. The PCR products were further amplified with primer pair E1011/E1012 using overlapping PCR, digested with XbaI and SalI and inserted into pEx18Km to generate pEx18Km-ΔVirE3::S11(522-541). pEx18Km-ΔVirE3::S11(522-541) was then introduced into *A. tumefaciens* strain EHA105*virE3::GFP11* to generate EHA105*virE3::GFP11*ΔVirE3(522-541).

#### EHA105ΔVirE3(522-541,598-644) and Its Derivatives

To generate *A. tumefaciens* strains EHA105ΔVirE3(522-541,598-644) and EHA105*virE2::GFP11*ΔVirE3(522-541,598-644), the flanking sequences were amplified from the total DNA of *A. tumefaciens* strain EHA105ΔVirE3(598-644) with primer pair E1007/E1008 or E1009/E1010. The PCR products were further amplified with primer pair E1011/E1012 using overlapping PCR, digested with XbaI and SalI and inserted into pEx18Km to generate pEx18Km-ΔVirE3(522-541,598-644). pEx18Km-ΔVirE3(522-541,598-644) was then introduced into *A. tumefaciens* strain EHA105ΔVirE3(598-644) or EHA105*virE2::GFP11*ΔVirE3(598-644) to generate EHA105ΔVirE3(522-541,598-644) or EHA105*virE2::GFP11*ΔVirE3(522-541,598-644), respectively. To generate *A. tumefaciens* strain EHA105*virE3::GFP11*ΔVirE3(522-541,598-644), the flanking sequences were amplified from the total DNA of *A. tumefaciens* strain EHA105*virE3::GFP11*ΔVirE3(598-644) with primer pair E1007/E1008 or E1009/E1010. The PCR products were further amplified with primer pair E1011/E1012 using overlapping PCR, digested with XbaI and SalI and inserted into pEx18Km to generate pEx18Km-ΔVirE3::S11(522-541,598-644). pEx18Km-ΔVirE3::S11(522-541,598-644) was then introduced into *A. tumefaciens* strain EHA105*virE3::GFP11*ΔVirE3(598-644) to generate EHA105*virE3::GFP11*ΔVirE3(522-541,598-644).

### Plasmid Construction

#### sfCherry1–10 Expression Plasmid

To generate the binary plasmid psfCherry1–10 expression sfCherry1–10 on T-DNA, the sfCherry3C1–10 coding sequence was amplified with primer pair P1001/P1002, digested with XbaI and BamHI and inserted into pXY01. The expression of sfCherry1–10 was under control of a CaMV 35S promoter on T-DNA. To generate the binary plasmid pQH308GR expressing both GFP1–10 and sfCherry1–10 on T-DNA, the sfCherry3C1–10 coding sequence was amplified with primer pair P1001/P1002, digested with XbaI and BamHI and inserted into pQH308A to replace the DsRed coding sequence. The expression of sfCherry1–10 was under control of a CaMV 35S promoter on T-DNA.

#### Yeast Two-Hybrid Plasmids

The VirE3(510-551) coding sequence was amplified from the total DNA of *A. tumefaciens* strain EHA105 with primer pair P1003/P1004, digested with BamHI and XhoI and inserted into pGADT7 to generate pGADT7-VirE3(510-551). The VirE3(596-648) coding sequence was amplified from the total DNA of *A. tumefaciens* strain EHA105 with primer pair P1005/P1006, digested with BamHI and XhoI and inserted into pGADT7 to generate pGADT7-VirE3(596-648). The VirE3(649-672) coding sequence was amplified from the total DNA of *A. tumefaciens* strain EHA105 with primer pair P1007/P1008, digested with BamHI and XhoI and inserted into pGADT7 to generate pGADT7-VirE3(649-672).

#### *In Vitro* Pull-Down Plasmids

The VirE3(510-551) coding sequence was amplified from the total DNA of *A. tumefaciens* strain EHA105 with primer pair P1009/P1004, digested with BamHI and XhoI and inserted into pMAL-c2x to generate pMAL-VirE3(510-551). The VirE3(596-648) coding sequence was amplified from the total DNA of *A. tumefaciens* strain EHA105 with primer pair P1010/P1006, digested with BamHI and XhoI and inserted into pMAL-c2x to generate pMAL-VirE3(596-648). The VirE2 coding sequence was amplified from the total DNA of *A. tumefaciens* strain EHA105 with primer pair P1011/P1012, digested with XbaI and KpnI and inserted into pRSET-A to generate pRSET-E2.

### Agroinfiltration

*N. benthamiana* wild-type and transgenic line Nb308A plants were used in agroinfiltration experiments as described previously (Li et al., 2014). Briefly, *A. tumefaciens* cells were firstly inoculated into LB medium to grow overnight; the cultured bacterial cells were harvested and diluted in fresh LB medium to OD_600_ = 0.1 and grown for additional 6–8 h. The bacteria were then resuspended in H_2_O and infiltrated into the underside of fully expended *N. benthamiana* leaves using a syringe. The infiltrated plants were placed at 22°C in a 16-h light/8-h dark photoperiod.

### Transient Expression in *N. benthamiana*

For the transient transformation assay, wild-type *N. benthamiana* leaves were infiltrated with *A. tumefaciens* strains containing pmC13-Reverse, which encodes mCherry within the T-DNA region. The bacterial cell suspensions were infiltrated into plant leaves at a concentration as stated in the figure legends. Images were obtained 2 days after agroinfiltration and used for fluorescence intensity calculation.

### Detection of VirE2 and VirE3 in Plants

Detection of *Agrobacterium*-delivered VirE2 and VirE3 was achieved using the split-GFP and split-sfCherry systems. GFP11 and sfCherry11 were used to label the effectors inside the bacterial cells. The labeled effectors were delivered into *N. benthamiana* leaf cells through agroinfiltration. Stable expression of GFP1–10 inside the plant cells was achieved using the transgenic line Nb308A. Transient expression of GFP1–10 and sfCherry1–10 was achieved using *A. tumefaciens* strains containing the binary plasmid pGFP1–10 or pQH308GR. Confocal microscopy was used to detect GFP_comp_ and sfCherry_comp_ signals at 2 days after agroinfiltration.

### Yeast-Two Hybrid Assay

Yeast-two hybrid assay was performed following the user manual (Clontech). Briefly, constructed plasmids were introduced into yeast strain AH109 through the lithium acetate-mediated transformation approach and the transformants were selected on SD/–Leu/–Trp plates. The transformed yeast cells were then cultured overnight in SD/–Leu/–Trp liquid medium. The cultured yeast cells were washed twice with H_2_O and resuspended in H_2_O to OD_600_ = 1. A series of dilutions (1/10) of the resuspended cells were then dropped onto the SD/–Leu/–Trp/ and SD/–Ade/–His/–Leu/–Trp/ plates and incubated at 30°C.

### Pull-Down Assay

BL21(DE3) *Escherichia coli* strain was used in protein expression. Briefly, *E. coli* strains containing the corresponding plasmids were grown to mid-log phase (OD_600_ = 0.6), isopropyl-β-D-thiogalactoside (IPTG) was then added into the cell cultures at a final concentration of 1 mM; the cells were allowed to grow at 28°C for 5 h. Bacterial cells were then resuspended in lysis buffer (50 mM tris-HCl, 100 mM NaCl, pH 7.5) and lysed by sonication. The supernatant of bait proteins (MBP or MBP-tagged proteins) was incubated with 80 μl of amylose resin (New England Biolabs) at 4°C for 4 h. The column was then washed five times with the lysis buffer. After that, the supernatant of the prey proteins (VirE2) was added into the column and incubated on a rotator at 4°C overnight. The column was then washed five times with the lysis buffer, and captured proteins were eluted with the lysis buffer containing 10 mM maltose. The eluted proteins were used for immunoblotting with antibodies against MBP or VirE2.

### Sequences of VirE3 and Its Homologs for Alignment

*Agrobacterium tumefaciens* (NCBI accession number: WP_012478092.1), *Agrobacterium arsenijevicii* (NCBI accession number: WP_045024006.1), *Rhizobium rubi* (NCBI accession number: GAK72198.1), *Agrobacterium vitis* (NCBI accession number: WP_012649040.1), *Agrobacterium larrymoorei* (NCBI accession number: WP_027676208.1), *Rhizobium etli* (NCBI accession number: AAD55076.1), *Agrobacterium rhizogenes* (NCBI accession number: WP_012476046.1), *Rhizobium mesoamericanum* (NCBI accession number: CCM79810.1), *Mesorhizobium plurifarium* (NCBI accession number: WP_041010510.1), *Rhizobium leguminosarum* (NCBI accession number: WP_011654520.1) and *Sinorhizobium medicae* (NCBI accession number: WP_018009501.1), encoding VirE3 and its homologs, were used for the alignment.

### Confocal Microscopy

A PerkinElmer UltraView Vox Spinning Disk system with electron-multiplying charge-coupled device cameras was used for confocal microscopy. All images were captured at 2 days post agroinfiltration and processed by Volocity 3D Image Analysis Software 6.2.1. Images for the transient transformation assay were obtained 2 days after agroinfiltration under confocal microscopy with an Olympus UPL SAPO 10 × numerical aperture (NA) 0.40 objective. Detection of *Agrobacterium*-delivered VirE2 and VirE3 were performed using an Olympus UPLSAPO 60 × NA 1.20 water-immersion objective.

### Quantification of Fluorescence Intensity

Fluorescence intensity was measured using ImageJ (https://imagej.nih.gov/ij/).

#### Fluorescence Intensity of VirE2-GFP_comp_

To calculate VirE2-GFP_comp_ signals associated with host cell borders, two confocal images of the same imaging field were taken sequentially at 1-min intervals. The “VirE2-GFP_comp_ signals associated with host cell borders” were selected as VirE2-GFP_comp_ signals that did not have spatial change during the time period and had direct contact with the host cell borders (illustrated by DsRed). All the VirE2-GFP_comp_ signals associated with host cell borders in each single imaging field were then calculated using ImageJ by deducting fluorescence intensity of the background (the surrounding areas) from the fluorescence intensity of selected areas.

#### Fluorescence Intensity of VirE3-GFP_comp_

As only plasma membrane localization of VirE3 was observed in our experimental conditions, all the detected VirE3-GFP_comp_ signals were considered as “VirE3-GFP_comp_ signals associated with host cell borders.” All the VirE3-GFP_comp_ signals associated with host cell borders in each single imaging field were calculated using ImageJ by deducting fluorescence intensity of the background (the surrounding areas) from the fluorescence intensity of selected areas.

#### Fluorescence Intensity of Transiently Expressed mCherry

Confocal images from the leaf areas without agroinfiltration treatment were used to calculate the fluorescence intensity of the background (plastid autofluorescence) using ImageJ. Fluorescence intensity of transiently expressed mCherry in each single imaging field was calculated using ImageJ by deducting fluorescence intensity of the background from the fluorescence intensity of the whole image.

### Statistical Analysis

Quantitative data are presented as means ± standard deviation (SD) from at least three independent experiments. When appropriate, statistical differences between groups were analyzed using an unpaired Student's *t*-test. Differences were considered significant at *P* < 0.01.

## Results

### *Agrobacterium*-Delivered VirE2 and VirE3 Co-Localize With Each Other at the Host Cell Border

A split-GFP strategy was adopted to visualize *Agrobacterium*-delivered VirE2 and VirE3 during the AMT process as described previously (Li et al., 2014, 2018; Li and Pan, 2017; Yang et al., 2017). The split-GFP system is composed of two non-fluorescent fragments of GFP: GFP1–10 (containing the β-strands 1–10 of GFP) and GFP11 (containing the β-strand 11 of GFP), which can bind to each other spontaneously and restore the fluorescence (Cabantous et al., 2005). The 16 amino-acid GFP11 was used to label the effector proteins inside bacterial cells and the GFP1–10 was expressed in host cells; spontaneous complementation of the two fragments occurred upon effector translocation and resulted in fluorescent labeling of the effectors in the host cell.

**Figure 1:**
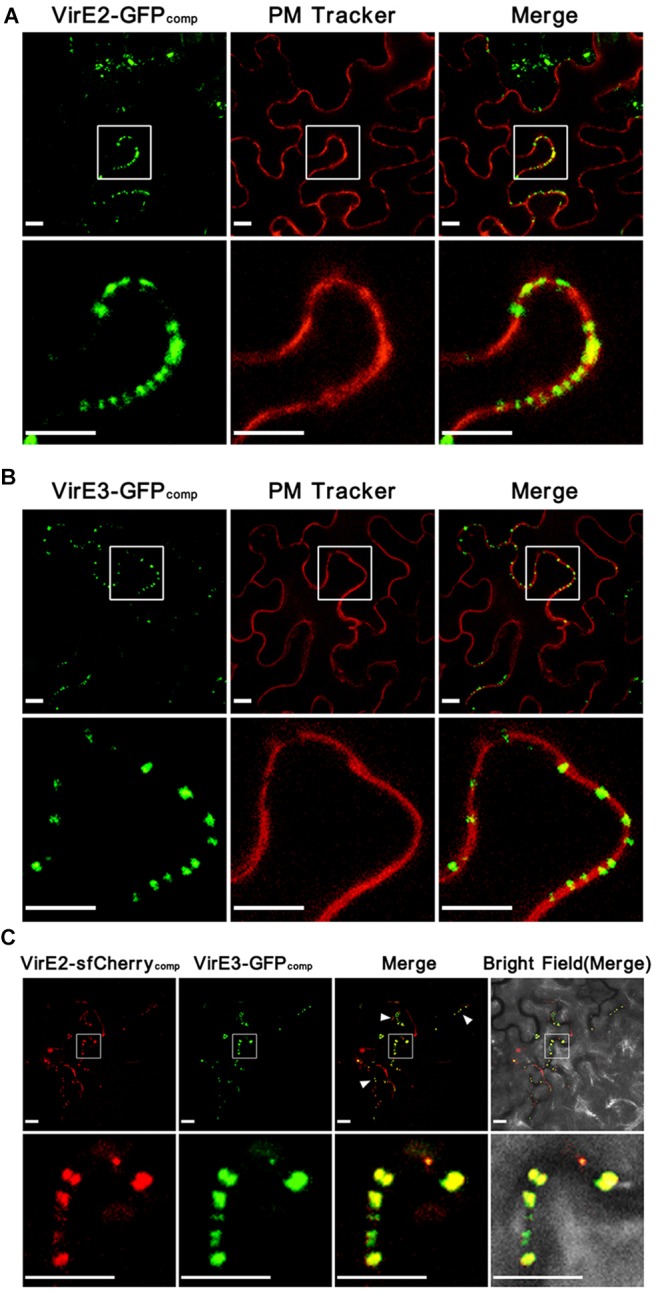
*Agrobacterium*-delivered VirE2 and VirE3 co-localize with each other at the cytoplasmic side of the host plasma membrane. **(A)** Accumulation of *Agrobacterium*-delivered VirE2 on the host plasma membrane. Wild-type *N. benthamiana* leaves were infiltrated with evenly mixed *A. tumefaciens*, EHA105*virE2::GFP11* containing a binary plasmid pGFP1–10 (expressing GFP1–10 on T-DNA) and EHA105*virE2::GFP11* containing a binary plasmid pm-rb (expressing a plasma membrane [PM] tracker on T-DNA). A representative image is shown (upper panel). The boxed areas are enlarged to highlight the punctate structures of VirE2 on the host plasma membrane (lower panel). Scale bars, 10 μm. **(B)** Accumulation of *Agrobacterium*-delivered VirE3 on the host plasma membrane. Wild-type *N. benthamiana* leaves were infiltrated with evenly mixed *A. tumefaciens*, EHA105*virE3::GFP11* containing a binary plasmid pGFP1–10 and EHA105*virE3::GFP11* containing a binary plasmid pm-rb. A representative image is shown (upper panel). The boxed areas are enlarged to highlight the punctate structures of VirE3 on the host plasma membrane (lower panel). Scale bars, 10 μm. **(C)** VirE2 and VirE3 co-localize with each other at the host cell border. Wild-type *N. benthamiana* leaves were infiltrated with *A. tumefaciens* strain EHA105-VirE2::sfCherry11-VirE3::GFP11 containing a binary plasmid pQH308GR (expressing GFP1–10 and sfCherry1–10 on T-DNA). A representative image is shown (upper panel). Arrowheads point to the co-localizations of VirE2 and VirE3 at the host cell border. The boxed areas are enlarged to highlight the punctate structures of VirE2 and VirE3 at the host cell border (lower panel). Scale bars: 10 μm.

To detect *Agrobacterium*-delivered VirE2 and VirE3, the GFP11-tagged strain EHA105*virE2::GFP11* or EHA105*virE3::GFP11* containing a binary plasmid pGFP1-10 (expressing GFP1–10 on T-DNA) was infiltrated into the leaves of wild-type *Nicotiana benthamiana* plants. As shown in (Figure 1), *Agrobacterium*-delivered VirE2 (Figure 1A) and VirE3 (Figure 1B) could co-localize with the plant plasma membrane, which was indicated by transient expression of a plant plasma membrane tracker (Nelson et al., 2007). This indicates that both VirE2 and VirE3 may target the host cellular membrane after secretion through the T4SS.

However, it is not clear whether VirE2 and VirE3 are spatially associated with each other on the host plasma membrane, since only one bipartite labeling system was available in previous studies (Li et al., 2014, 2018; Li and Pan, 2017). To further investigate this, we combined the split-GFP system together with a newly developed split-sfCherry system (Kamiyama et al., 2016; Feng et al., 2017, 2019). Similar as the split-GFP strategy, the split-sfCherry system is also composed of two non-fluorescent fragments of the superfolder Cherry (sfCherry): sfCherry1–10 (containing the β-strands 1–10 of sfCherry) and sfCherry11 (containing the β-strand 11 of sfCherry), which could restore the fluorescence upon spontaneous binding (Kamiyama et al., 2016). In this study, the split-GFP strategy was used to label *Agrobacterium*-delivered VirE3 as described (Li et al., 2018). Meanwhile, the split-sfCherry strategy was adopted to label *Agrobacterium*-delivered VirE2 by inserting the coding sequence of the 18 amino-acid sfCherry11 at the permissive site of *virE2* (Zhou and Christie, 1999) on the Ti plasmid.

To ensure that the sfCherry11 tag did not interfere with the function of VirE2, a transient transformation assay, based on transient expression of mCherry from the T-DNA, was conducted on *N. benthamiana* leaves to test the function of VirE2-sfCherry11. The *A. tumefaciens* strain EHA105-VirE2::sfCherry11-VirE3::GFP11, which expresses sfCherry11-labeled VirE2 and GFP11-labeled VirE3, containing a binary plasmid pmC13-Reverse was infiltrated into the wild-type *N. benthamiana* leaves; the untagged strains EHA105, EHA105ΔVirE2 and EHA105ΔVirE3 were used as the controls. Deletion of either *virE2* or *virE3* decreased the transient transformation efficiency; in contrast, the sfCherry11-labeled VirE2 and GFP11-labeled VirE3 functioned similarly as the untagged proteins inside the plant cell in the transient transformation assay (Figure S1). These indicate that sfCherry11 and GFP11 tags do not affect the protein function and thus are suitable for labeling of these effectors.

*A. tumefaciens* strain EHA105-VirE2::sfCherry11-VirE3::GFP11 was then infiltrated into *N. benthamiana* leaves to visualize *Agrobacterium*-delivered VirE2 and VirE3 inside host cells. As shown in (Figure 1C), *Agrobacterium*-delivered VirE2 and VirE3 were observed to co-localize with each other at the host cell border at two days post agroinfiltration. As GFP and mCherry have similar structures, we also tested whether the two labeling systems could cross-complement with each other. As shown in (Figure S2), no fluorescence signal for VirE2 could be detected using *A. tumefaciens* strain EHA105*virE2::sfCherry11* containing a binary plasmid pGFP1-10 as the control; and no fluorescence signal for VirE3 could be detected using *A. tumefaciens* strain EHA105*virE3::GFP11* containing a binary plasmid psfCherry1-10 (expressing sfCherry1–10 on T-DNA) as the control, demonstrating that these two labeling systems worked as expected and no cross-complementation occurred. These results indicate that VirE2 and VirE3 associate with each other on the host plasma membrane immediately after the translocation.

Since both GFP1–10 and sfCherry1–10 were expressed by T-DNA, they were supposed to be present inside the host cell. When they meet the corresponding partners delivered from the bacterial cells, the complementary fluorescence signals should be formed in the host cytoplasm. Therefore, our results suggested that VirE2 and VirE3 associated with each other on the cytoplasmic side of the host plasma membrane upon their delivery into the host cell. This is also supported by previous studies showing that *Agrobacterium*-delivered VirE2 was present in the cytosolic side of host endoplasmic reticulum (Yang et al., 2017).

### VirE3 Contains Two Putative VirE2-Interacting Domains at the C-Terminus

It was shown that the C-terminal domain of VirE3 could interact with VirE2 in the yeast two-hybrid assay (Li et al., 2018). However, deletion of this domain of VirE3 only decreased VirE2 accumulation at the host cell border, while no VirE2 accumulation on the cytoplasmic side of the host plasma membrane could be observed from the *virE3* deletion mutant (Li et al., 2018), indicating that other domain(s) of VirE3 may also be involved in VirE2 retention on the host plasma membrane.

**Figure 2:**
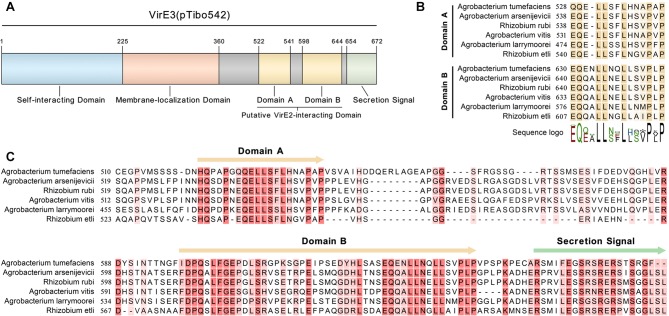
VirE3 contains two putative VirE2-interacting domains at the C-terminus. **(A)** Schematic representation of the functional domains in VirE3 (pTibo542). The relevant amino acid positions are indicated. **(B)** Sequence alignment of the C-terminus of *A. tumefaciens* VirE3 and its homologs from *Agrobacterium* and rhizobia species. **(C)** Partial sequence alignment of the two putative VirE2-interacting domains of *A. tumefaciens* VirE3 and its homologs from *Agrobacterium* and rhizobia species. Sequence alignments were carried out using the CLC Genomics Workbench software.

To identify other possible VirE2-interacting domain(s) of VirE3, we performed a sequence alignment with VirE3 homologs from various species of *Agrobacterium* and the rhizobia group. As shown in (Figure S3), the N-terminal region and the middle region of VirE3 are conserved in all the bacteria species, which have been shown to be involved in VirE3 self-interacting and membrane-localization in host cells, respectively (Li et al., 2018). Interestingly, there are two conserved domains (domain A and domain B) at the C-terminus of VirE3 (Figures 2A,B), which are only conserved in the bacteria species that contain genes encoding VirE2 homologs (Figure S3), suggesting that these two domains may be related to the function of VirE2. Sequence alignment also revealed that the C-terminal parts of these two domains had high similarities (Figure 2C), indicating that they may both be involved in VirE2 interaction.

### The Two Conserved Domains at VirE3 C-Terminus Interact With VirE2 *in vitro*

**Figure 3:**
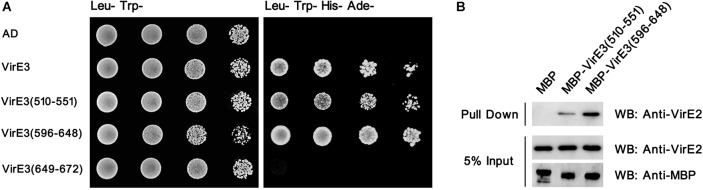
The two conserved domains at the C-terminus of VirE3 interact with VirE2 *in vitro*. **(A)** Yeast two-hybrid assay for VirE2 and VirE3. The C-terminal parts of VirE3 containing the putative VirE2-interacting domain A (amino acids 510-551), VirE2-interacting domain B (amino acids 596–648) and the secretion signal (amino acids 649-672) were cloned into pGADT7 (AD). The AD constructs were co-transferred into yeast strain AH109 together with pGBKT7-VirE2. The transformed cells were grown on SD medium minus tryptophan and leucine (Leu-Trp-), and SD medium minus tryptophan, leucine, histidine and adenine (Leu-Trp-His-Ade-). The empty AD or pGADT7-VirE3 was used as the negative or positive control, respectively. **(B)** Maltose-binding protein (MBP) pull-down assays for VirE2 and VirE3. MBP-tagged VirE2-interacting domain A or VirE2-interacting domain B was used as a bait and VirE2 was used as a prey. MBP alone was used as the control. Protein detections were carried out by western blots (WB) using antibodies as indicated.

To examine that whether domain A and domain B could interact with VirE2 *in vitro*, the yeast two-hybrid assay was performed. As shown in Figure 3A, both domain A and domain B of VirE3 could interact with VirE2 in yeast cells. In contrast, the last 24 amino acids, which have been shown to contain the secretion signal of VirE3, did not interact with VirE2 in the yeast two-hybrid assay (Figure 3A). These results suggest that the two putative VirE2-interacting domains and the secretion signal of VirE3 are located at distinct regions and do not overlap with each other.

To further verify these interactions, we performed pull-down assays using maltose-binding protein (MBP)-tagged domain A or domain B as a bait and VirE2 as a prey. Pull-down and immunoblotting assays showed that VirE2 could be precipitated by both MBP-tagged domain A and domain B, but not MBP alone, indicating that both domain A and domain B of VirE3 could interact with VirE2 *in vitro* (Figure 3B).

### The Two VirE2-Interacting Domains of VirE3 Are Involved in VirE2 Retention on the Host Plasma Membrane

**Figure 4:**
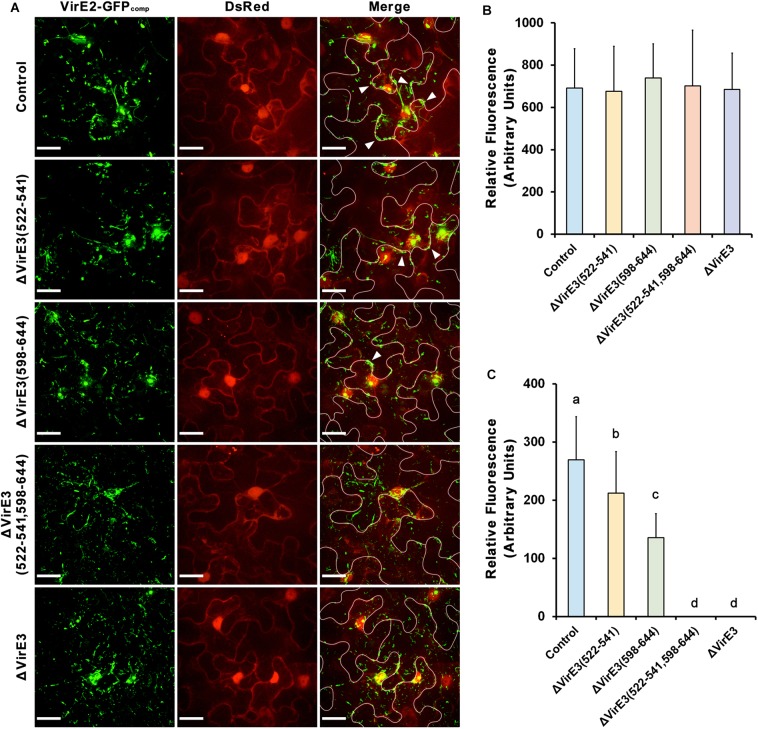
Deletions of VirE2-interacting domains of VirE3 affect VirE2 accumulation at the host cell border. **(A)** *N. benthamiana* (Nb308A) leaves were infiltrated with *A. tumefaciens* EHA105*virE2::GFP11* (control), EHA105*virE2::GFP11*ΔVirE3(522-541), EHA105*virE2::GFP11*ΔVirE3(598-644), EHA105*virE2::GFP11*ΔVirE3(522-541,598-644) or EHA105*virE2::GFP11*ΔVirE3. White lines are added to indicate borders between leaf epidermal cells. Arrowheads point to VirE2 aggregates at the plant cell border. Scale bars, 20 μm. **(B)** The total fluorescence intensity of VirE2-GFP_comp_ signals was measured in each image. Data are presented as means ± SDs of *n* = 20 independent samples. **(C)** The fluorescence intensity of VirE2-GFP_comp_ signals associated with host cell borders was measured in each image. Data are presented as means ± SDs of *n* = 20 independent samples. *p* < 0.01.

Considering that VirE2 and VirE3 are translated from the same polycistronic mRNA inside the bacterial cell, the interaction between them implies their functional association during the transformation. To further investigate this, mutants with single and double deletion of VirE2-interacting domains of VirE3 were generated. The mutant strains were then infiltrated into the leaves of transgenic *N. benthamiana* (Nb308A) plants, which expressed both GFP1–10 and DsRed, to track *Agrobacterium*-delivered VirE2 (Figure 4A).

Total fluorescence of VirE2-GFP_comp_ signals inside the plant cells were measured at 2 days post agroinfiltration. Our results showed that deletion of VirE2-interacting domains or full length VirE3 did not affect the total fluorescence intensities of VirE2-GFP_comp_ signals inside host cells (Figures 4A,B), indicating that translocation of VirE2 was not affected in these mutants.

In contrast, the amount of VirE2 accumulated at the host cell border decreased from the VirE2-interacting domain deletion mutants as compared to the control group (Figures 4A,C). Deletion of VirE2-interacting domain A in VirE3 caused a minor decrease in VirE2 accumulation at the cytoplasmic side of the host cell border, while deletion of VirE2-interacting domain B had a stronger effect. Moreover, double deletion of VirE2-interacting domains in VirE3 abolished the accumulation of VirE2 at the host cellular membrane similarly as that of the *virE3* deletion mutant. Compared with the control group (Supplementary Movie S1), deletion of the two VirE2-interacting domains (Supplementary Movie S2) or full length *virE3* (Supplementary Movie S3) caused abnormal distribution of VirE2 that it could not stay stably on the host plasma membrane. These data indicate that redundant function may be shared for the two VirE2-interacting domains of VirE3.

**Figure 5:**
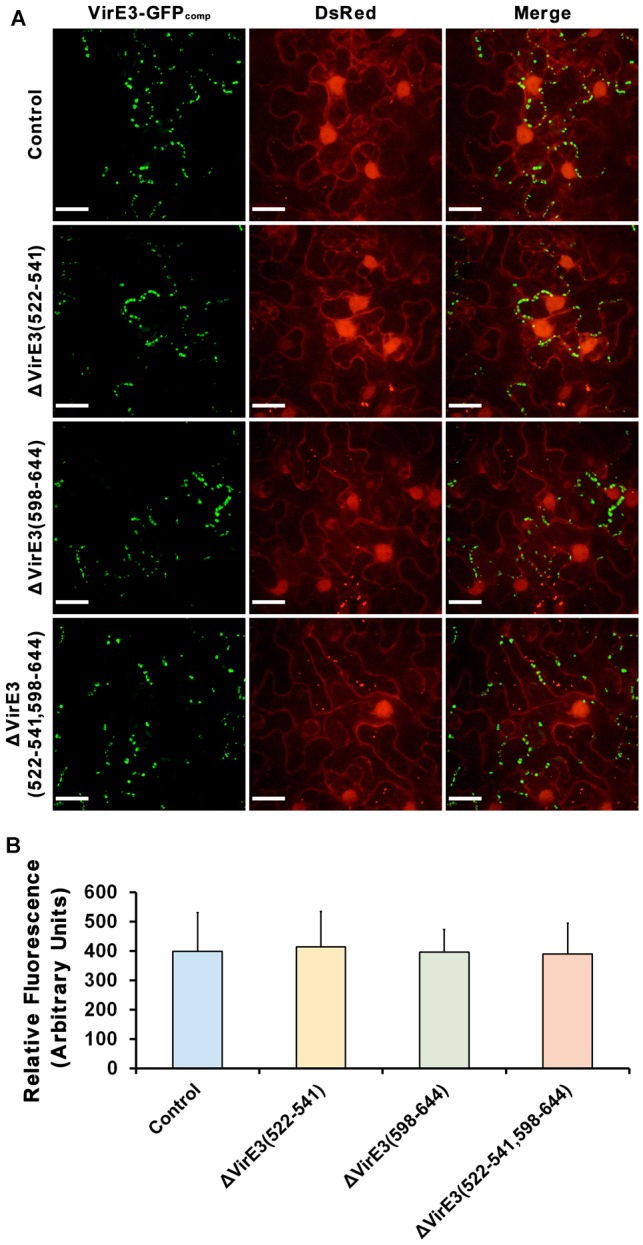
Deletions of VirE2-interacting domains of VirE3 do not affect VirE3 accumulation at the host cell border. **(A)** *N. benthamiana* (Nb308A) leaves were infiltrated with *A. tumefaciens* EHA105*virE3::GFP11* (control), EHA105*virE3::GFP11*ΔVirE3(522-541), EHA105*virE3::GFP11*ΔVirE3(598-644) or EHA105*virE3::GFP11*ΔVirE3(522-541,598-644). Scale bars, 20 μm. **(B)** The fluorescence intensity of VirE3-GFP_comp_ signals was measured in each image. Data are presented as the mean ± SD of *n* = 20 independent samples.

Considering that the two VirE2-interacting domains are spatially close to the secretion signal of VirE3 at its C-terminus, we also examined *Agrobacterium*-delivered VirE3 from the mutants to ensure that deletion of VirE2-interacting domains did not affect the secretion of VirE3. Mutants with single and double deletions of VirE2-interacting domains in VirE3 were generated from *A. tumefaciens* strain EHA105*virE3::GFP11*. These mutant strains were then infiltrated into the leaves of transgenic *N. benthamiana* (Nb308A) to localize *Agrobacterium*-delivered VirE3. Similar VirE3 localizations were observed from the mutant and the control strains (Figures 5A,B), suggesting that deletion of VirE2-interacting domains in VirE3 did not affect VirE3 secretion and aggregation on the host plasma membrane. Taken together, these results indicate that both the two VirE2-interacting domains of VirE3 function in retention of VirE2 on the cytoplasmic side of the host plasma membrane during the AMT process.

### The Two VirE2-Interacting Domains of VirE3 Are Important for the Transformation Process

Deletion of *virE3* caused the truncations of T-DNA during the AMT process and decreased transformation efficiency in the transient transformation assay of *N. benthamiana* leaves (Li et al., 2018). To further confirm the importance of these two VirE2-interacting domains in the transformation, single and double deletions of VirE2-interacting domains in VirE3 were generated from *A. tumefaciens* strain EHA105. The transient transformation abilities of these mutant strains on *N. benthamiana* leaves were then examined.

**Figure 6:**
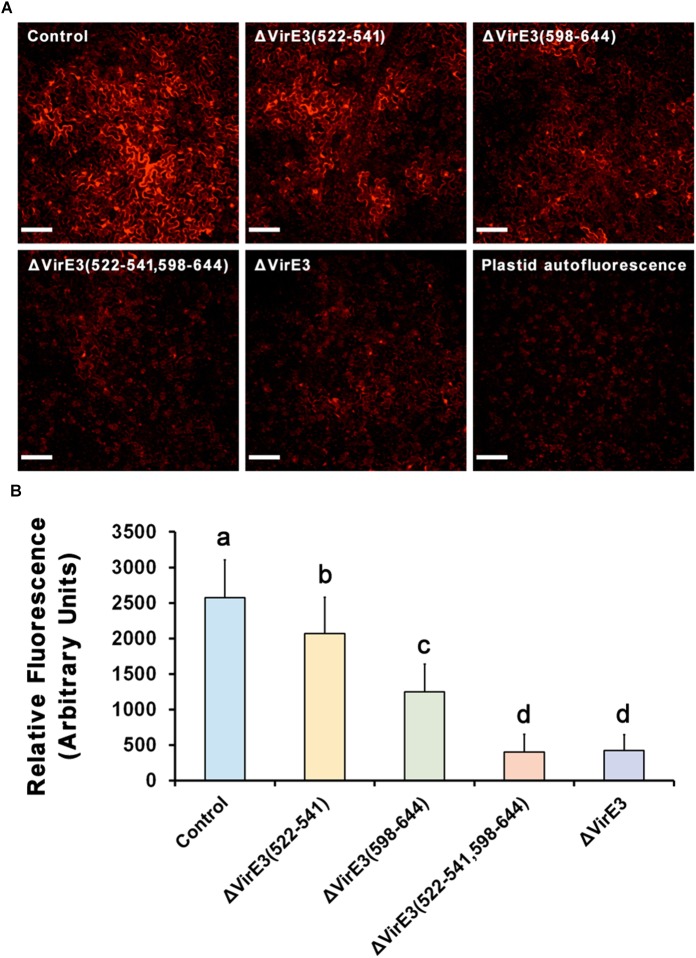
Deletions of VirE2-interacting domains of VirE3 attenuate the transient transformation efficiency of *A. tumefaciens*. **(A)** Wild-type *N. benthamiana* leaves were infiltrated with *A. tumefaciens* EHA105 (control), EHA105ΔVirE3(522-541), EHA105ΔVirE3(598-644), EHA105ΔVirE3(522-541,598-644) or EHA105ΔVirE3 containing a binary plasmid pmC13-Reverse (expressing free mCherry under the CaMV 35S promoter on T-DNA). Scale bars, 100 μm. **(B)** The fluorescence intensity of transiently expressed mCherry was measured in each image. Data are presented as means ± SDs of *n* = 30 independent samples. *p* < 0.01.

As shown in (Figure 6), all three mutants showed decreased transient transformation capabilities as compared with the wild-type strain. Deletion of VirE2-interacting domain B in VirE3 led to a more significant decrease in the transformation efficiency as compared with the deletion of VirE2-interacting domain A. This suggests that the VirE2-interacting domain B has a more important role during the transformation than the VirE2-interacting domain A. This is also consistent with our above observations that the VirE2-interacting domain B-deletion mutant was less competent to retain VirE2 on the host cellular membrane (Figures 4A,C). Moreover, the double deletion mutant showed a similar efficiency of transient transformation as the *virE3* deletion mutant (Figures 6A,B), indicating that retention of VirE2 on the host cellular membrane is the main function of VirE3 during the AMT process. Similar results have also been observed by using different concentration of bacterial cells (Figure S4). Taken together, our results suggest that the two VirE2-interacting domains of VirE3 are required to retain VirE2 at the cytoplasmic side of the host cellular membrane in the transformation process, which may be important for T-DNA protection and T-complex assembly.

## Discussion

Effector proteins are the critical “players” in bacteria-host interactions. Direct labeling and tracking of these proteins inside host cells are important to understand their functions. The split-GFP system composed of two self-complementing fragments of GFP enables the spatiotemporal monitoring of bacterial secreted effectors in various host cells (Van Engelenburg and Palmer, 2010; Sakalis et al., 2014; Henry et al., 2017; Park et al., 2017; Tu et al., 2018). Recently, a new split-sfCherry system was generated using the sfCherry and opened up the possibility of multicolor imaging in effector protein studies (Kamiyama et al., 2016; Feng et al., 2017, 2019). In this paper, for the first time we have combined the split-GFP and split-sfCherry systems to directly visualize different effector proteins from the bacteria. A sfCherry3C1-10/11 tagging system (Feng et al., 2019) was used in our study due to its enhanced brightness and complementation efficiency.

Our results reveal that these two labeling system work compatibly in spatiotemporal tracking of *Agrobacterium*-delivered effector proteins VirE2 and VirE3, although the complemented sfCherry_comp_ shows lower fluorescence intensities than GFP_comp_, presumably due to less efficient complementation efficiency as reported (Feng et al., 2019). During bacteria-host interactions, different effectors may function cooperatively to achieve the same goal and they may be spatially and temporally associated inside the host cell; thus, the combination of the two self-associating split-fluorescent protein systems is an important and useful approach to label different effectors simultaneously.

VirE3 was reported to be not essential for AMT and deletion of *virE3* did not affect the transformation efficiency significantly (Kalogeraki et al., 2000; Garcia-Rodriguez et al., 2006). Here we show that VirE3 is required for full virulence of *Agrobacterium*. The different observations may result from different assays used for transformation efficiency calculation. In previous studies, the transformation abilities of *Agrobacterium* cells were evaluated based on the tumor formation on the plant leaves or stems, which may represent the overall results from each leaf or stem tissue. In contrast, we are calculating transient transformation efficiency of *Agrobacterium* strains based on the fluorescence signal intensities from individual host cells, which may be more sensitive to detect subtle differences between various mutants. Another possible explanation for the discrepancy may be the difference in the amounts of bacterial cells used for transformation. In our studies, the difference detected between the wild-type strain and the *virE3* deletion mutant could only be observed when the number of used bacterial cells was low; and the difference became less apparent when a high concentration of *Agrobacterium* was used for leaf infiltration. The high concentration probably saturated the transformation so that the difference became unapparent.

Previous studies showed that *Agrobacterium*-delivered VirE2 accumulated on the host plasma membrane first and targeted the host nucleus subsequently (Li and Pan, 2017). In contrast, we observed that VirE3 localized on the host plasma membrane exclusively during the whole transformation process. This suggests that VirE3 may mainly target the host plasma membrane and function at the early stage of the transformation rather than the subsequent steps involving VirE2.

By using the split-fluorescent protein systems, we show that *Agrobacterium*-delivered VirE2 and VirE3 co-localize with each other at the cytoplasmic side of the host cell border. Thus, VirE2 and VirE3 may only physically associate with each other temporally at the host cell entrance during the AMT process. VirE3 was reported to possess a conserved membrane-localization domain in the middle of the protein which was necessary to target the plant cellular membrane; deletion of *virE3* abolished the VirE2 accumulation at the host cell border, while inhibition of VirE2 secretion did not affect the VirE3 accumulation on the host plasma membrane (Li et al., 2018), indicating that VirE3 targeted the host plasma membrane first and retained VirE2 at the host cell entrance.

VirE3 was previously reported to localize to the plant nucleus (Lacroix et al., 2005; Niu et al., 2015). However, we have observed an exclusive localization of VirE3 on the host plasma membrane in *N. benthamiana* leaf cells under confocal microscopy. The discrepancy in VirE3 localization may result from the different labeling or expression approaches that may affect VirE3 localization. As the previous observations were based on transient expression of VirE3 from the bombardment or the protoplast, harsh treatment like bombardment or cell wall digestion during protoplast generation may artificially cause the relocation of VirE3, although this still needs further verification. Moreover, potential transcriptional activity of VirE3 has also been reported (Garcia-Rodriguez et al., 2006; Niu et al., 2015). Our present studies could not exclude the possibility that VirE3 may go to the host nucleus at a low level beyond the detection sensitivity of the confocal microscopy. However, our results show that double deletion of the two VirE2-interacting domains in VirE3 decreased the transformation efficiency to a level comparable to the *virE3* deletion mutant, indicating that interacting with VirE2 is the major function of VirE3 during AMT. Moreover, deletion of *virE3* could be extracellularly complemented by an excess amount of VirE2 in the transient transformation assay (Li et al., 2018), suggesting that VirE3 facilitated the transformation process mainly through VirE2. Thus, the potential transcriptional activity of VirE3 may only assist the transformation process in a subtle way and may not affect the transformation efficiency significantly.

Recently, a symbiotic bacterium *Rhizobium etli* has been shown to be able to transform plant cells, suggesting that the ability of genetic transformation of eukaryotic cells is not limited to *Agrobacterium* genus (Lacroix and Citovsky, 2016). Although *R. etli* does not harbor any T-DNA-like sequences, its p42a plasmid contains a complete set of *vir* genes, including a *virE3* homolog, which may be acquired from an ancestral bacterial species or via conjugation. VirE3 is a conserved protein from *Agrobacterium* and several rhizobia species including *R. etli*, suggesting its conserved function in these bacteria. As compared to the N-terminal regions, the C-terminal regions of these VirE3 homologs are much less conserved in *Agrobacterium rhizogenes* and rhizobia species, some of which lack the VirE2-interacting domains (Li et al., 2018). Interestingly, these bacterial species (e.g., *Agrobacterium rhizogenes, Rhizobium mesoamericanum, Mesorhizobium plurifarium, Rhizobium leguminosarum* and *Sinorhizobium medicae*) without the VirE2-interacting domains in VirE3 also lack the corresponding genes encoding VirE2 homologs. *A. rhizogenes* encodes two GALLs proteins that could substitute for *A. tumefaciens* VirE2 in T-DNA protection (Hodges et al., 2004, 2006, 2009), although they do not resemble each other. Thus, it would be of interest to determine the roles of VirE3 homologs lacking the VirE2-interacting domains in future.

VirE2 could form filamentous structures through self-interactions, thus the two tandem VirE2-interacting domains of VirE3 may facilitate the linkage of VirE2 from head to tail, which may further facilitate the coating of T-DNA at the host cell entrance. Although the two VirE2-interacting domains at VirE3 C-terminus show sequence similarities, they behave differently in VirE2 retention on the cytoplasmic side of the host plasma membrane. VirE2-interaction domain A is shorter in length than VirE2-interacting domain B (Figure 2B); VirE2-interaction domain A also showed weaker interactions with VirE2 *in vitro* (Figure 3). Deletion of VirE2-interacting domain B of VirE3 led to stronger effects both in VirE2 retention on the host plasma membrane and AMT efficiency, suggesting that these two VirE2-interacting domains may have different degrees of significance in the transformation. VirE2 is temporally retained at the cytoplasmic side of the host cellular membrane and targets the host nucleus subsequently (Li and Pan, 2017); thus, the two tandem VirE2-interacting domains with different VirE2-binding affinities may also function to facilitate the release of VirE2 from the host plasma membrane. Future studies are needed to investigate the cooperation between the two VirE2-interacting domains of VirE3 during the AMT process.

## Data Availability Statement

All datasets generated for this study are included in the article/Supplementary Material.

## Author Contributions

XL designed the study, performed the experiments, analyzed and interpreted the data, and wrote the initial manuscript. TZ and HT performed the experiments. SP supervised the project, interpreted the data, conceived the concept, and improved the manuscript.

## Conflict of Interest

The authors declare that the research was conducted in the absence of any commercial or financial relationships that could be construed as a potential conflict of interest.
