# Supplementary material for: Agrobacterium VirE3 Uses Its Two Tandem Domains at the C-Terminus to Retain Its Companion VirE2 on the Cytoplasmic Side of the Host Plasma Membrane
Source: Front Plant Sci. 2020 Apr 21;11:464. doi: 10.3389/fpls.2020.00464 (PMC7187210; doi:10.3389/fpls.2020.00464)
Supplement: Supplementary file 1 [file Data_Sheet_1.docx]

**Supplementary Material**

**
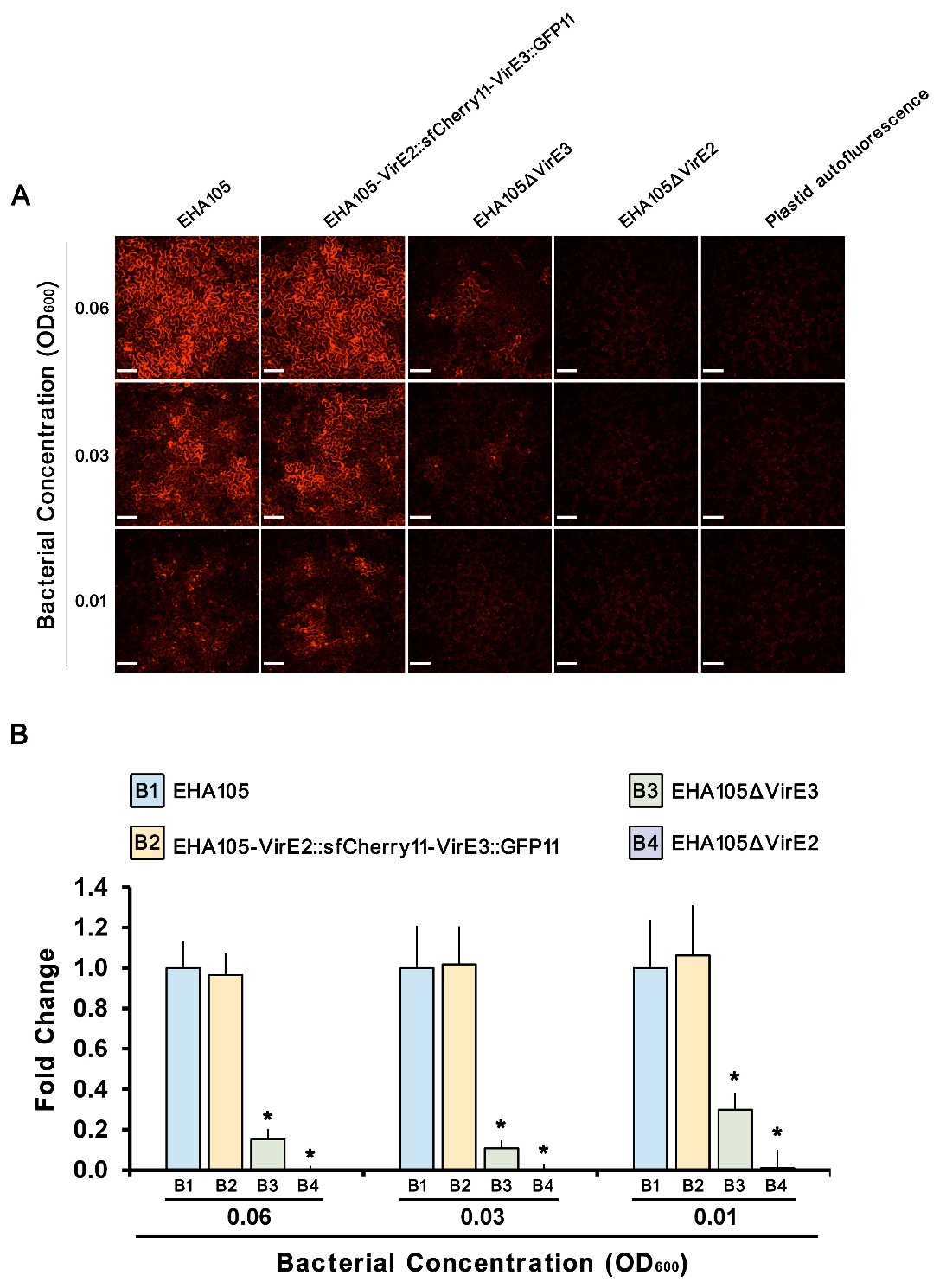
**

**Figure S1.** sfCherry11-labeled VirE2 and GFP11-labeled VirE3 function similarly like the untagged proteins inside the plant cell in the transient transformation assay. **(A)** Wild-type *N. benthamiana* leaves were infiltrated with different concentrations of *A. tumefaciens* cells EHA105, EHA105-VirE2::sfCherry11-VirE3::GFP11, EHA105ΔVirE3 or EHA105ΔVirE2 containing a binary plasmid pmC13-Reverse (expressing free mCherry under the CaMV 35S promoter on T-DNA). Scale bars, 100 μm. **(B)** The fluorescence intensity of transiently expressed mCherry was measured in each image. The intensity was normalized to the control group. Data are presented as means ± SDs of n = 30 independent samples. *p < 0.01.

**
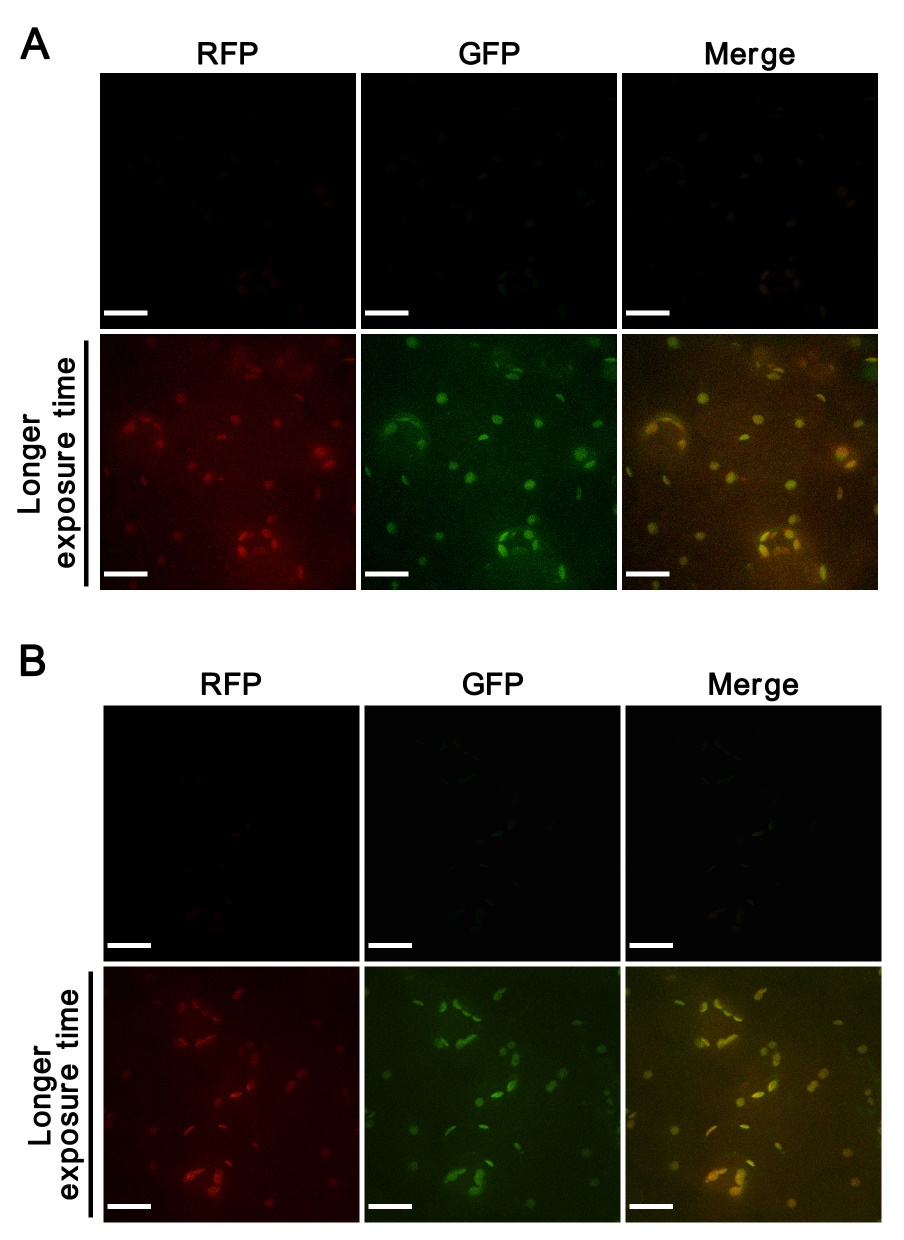
**

**Figure. S2.** Split-GFP and split-sfCherry systems do not interfere with each other. **(A)** Wild-type *N. benthamiana* leaves were infiltrated with *A. tumefaciens* strain EHA105*virE2::sfCherry11* containing a binary plasmid pGFP1-10 (expressing GFP1–10 on T-DNA), and no fluorescence signal for VirE2 could be detected at two days post agroinfiltration (upper panel). Longer exposure time was used for the same imaging field to detect the autofluorescence of plastids (lower panel). Scale bars, 20 μm. **(B)** Wild-type *N. benthamiana* leaves were infiltrated with *A. tumefaciens* strain EHA105*virE3::GFP11* containing a binary plasmid psfCherry1-10 (expressing sfCherry1–10 on T-DNA); and no fluorescence signal for VirE3 could be detected at two days post agroinfiltration (upper panel). Longer exposure time was used for the same imaging field to detect the autofluorescence of plastids (lower panel). Scale bars, 20 μm.

**
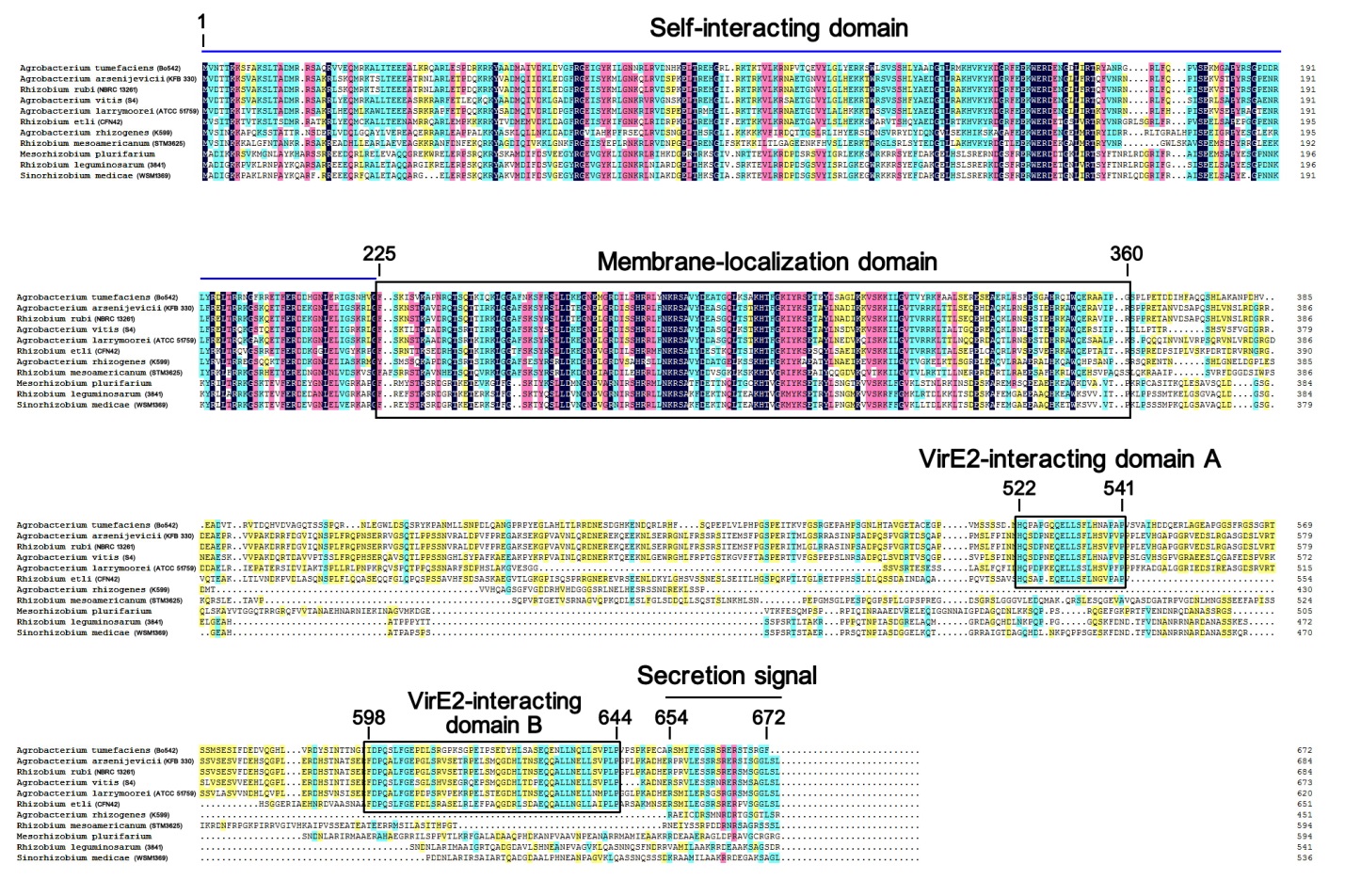
**

**Figure S3.** Sequence alignment of *Agrobacterium* VirE3 and its homologs from *Rhizobium/Agrobacterium* species. The amino acid sequence of VirE3 from *A. tumefaciens* (NCBI accession number: WP_012478092.1) was aligned with its homologs from *Agrobacterium arsenijevicii* (NCBI accession number: WP_045024006.1), *Rhizobium rubi* (NCBI accession number: GAK72198.1), *Agrobacterium vitis* (NCBI accession number: WP_012649040.1), *Agrobacterium larrymoorei* (NCBI accession number: WP_027676208.1), *Rhizobium* *etli* (NCBI accession number: AAD55076.1), *Agrobacterium rhizogenes* (NCBI accession number: WP_012476046.1), *Rhizobium mesoamericanum* (NCBI accession number: CCM79810.1), *Mesorhizobium* *plurifarium* (NCBI accession number: WP_041010510.1), *Rhizobium leguminosarum* (NCBI accession number: WP_011654520.1) and *Sinorhizobium medicae* (NCBI accession number: WP_018009501.1) using the DNAMAN software (Version 7.0.2.176). The self-interacting domain, membrane-localization domain, VirE2-interacting domains and the secretion signal motif are delineated. The relevant amino acid positions are indicated.


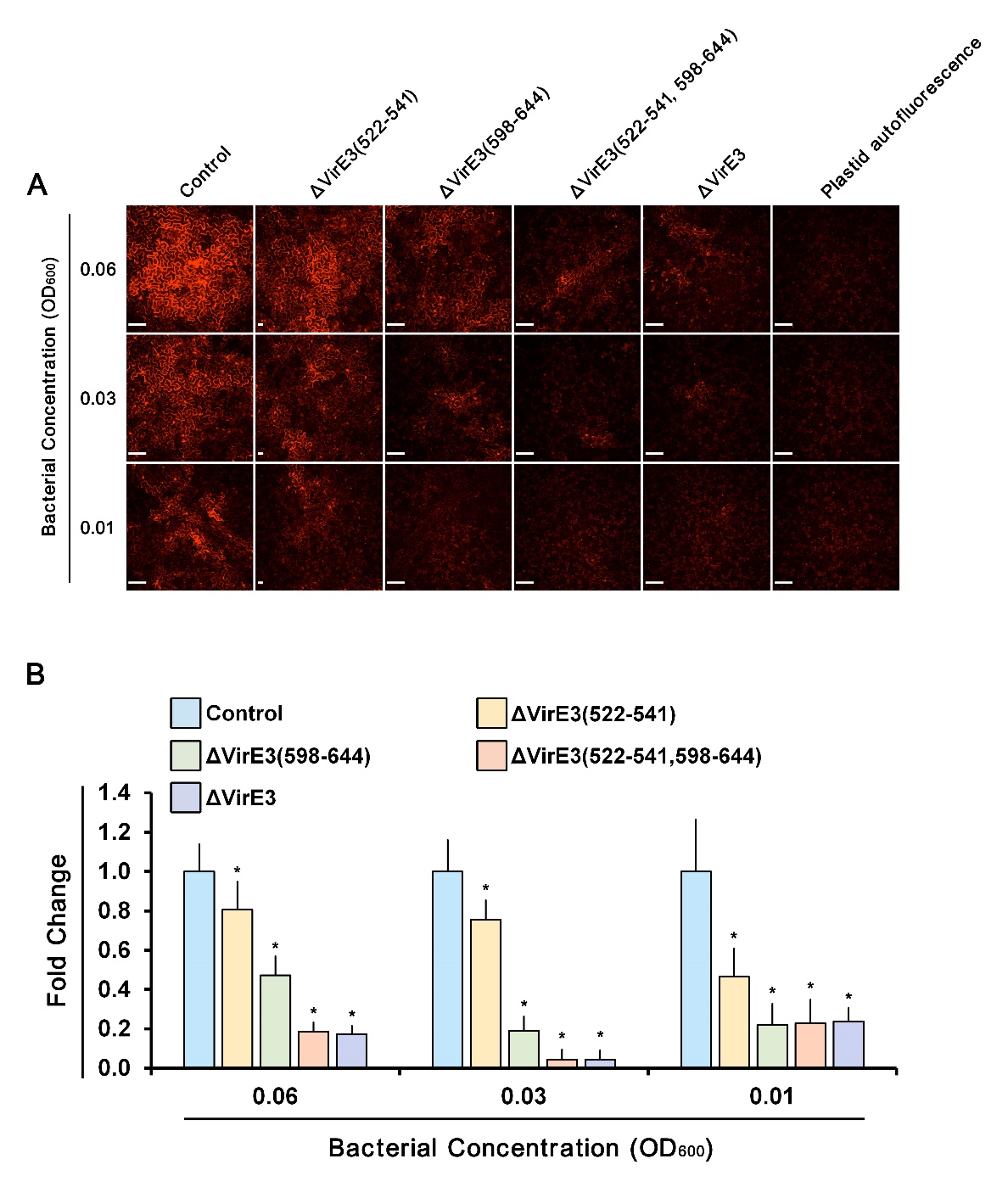


**Figure S4.** Deletions of VirE2-interacting domains of VirE3 attenuate the transient transformation efficiency of *A. tumefaciens*. **(A)** Wild-type *N. benthamiana* leaves were infiltrated with different concentrations of *A. tumefaciens* cells EHA105 (control), EHA105ΔVirE3(522-541), EHA105ΔVirE3(598-644), EHA105ΔVirE3(522-541,598-644) or EHA105ΔVirE3 containing a binary plasmid pmC13-Reverse (expressing free mCherry on T-DNA). Scale bars, 100 μm. **(B)** The fluorescence intensity of transiently expressed mCherry was measured in each image. The intensity was normalized to the control group. Data are presented as means ± SDs of n = 30 independent samples. *p < 0.01.

**Supplementary Movie legends**

**Movie S1.** Localization of VirE2-GFP_comp_ in host cells in the presence of wild-type VirE3. *N. benthamiana* (Nb308A) leaves were infiltrated with *A. tumefaciens* EHA105*virE2::GFP11*. The VirE2-GFP_comp_ signals were visualized under a confocal microscope at two days after agroinfiltration. DsRed indicates cellular structures. Scale bar, 20 μm.

**Movie S2.** Localization of VirE2-GFP_comp_ in host cells in the presence of VirE3 with double deletion of VirE2-interacting domains. *N. benthamiana* (Nb308A) leaves were infiltrated with *A. tumefaciens* EHA105*virE2::GFP11*ΔVirE3(522-541,598-644). The VirE2-GFP_comp_ signals were visualized under a confocal microscope at two days after agroinfiltration. DsRed indicates cellular structures. Scale bar, 20 μm.

**Movie S3.** Localization of VirE2-GFP_comp_ in host cells in the absence of VirE3. *N. benthamiana* (Nb308A) leaves were infiltrated with *A. tumefaciens* EHA105*virE2::GFP11*ΔVirE3. The VirE2-GFP_comp_ signals were visualized under a confocal microscope at two days after agroinfiltration. DsRed indicates cellular structures. Scale bar, 20 μm.

**Table S1.** *A. tumefaciens* Strains used in this study

| ***A. tumefaciens* Strains** | **Relevant characteristics** | **Source** |
| --- | --- | --- |
| EHA105 | C58 strain containing pTiBo542 without T-DNA | (Hood et al., 1993) |
| EHA105ΔVirE2 | EHA105 derivative, with *virE2* deleted from pTiBo542 | (Li et al., 2014) |
| EHA105ΔVirE3 | EHA105 derivative, with *virE3* deleted from pTiBo542 | (Li et al., 2018) |
| EHA105ΔVirE3(522-541) | EHA105 derivative, with the sequence encoding the VirE3 amino acids 522-541 deleted from the Ti plasmid | This Study |
| EHA105ΔVirE3(598-644) | EHA105 derivative, with the sequence encoding the VirE3 amino acids 596-644 deleted from the Ti plasmid | (Li et al., 2018) |
| EHA105ΔVirE3(522-541,598-644) | EHA105 derivative, with the sequence encoding the VirE3 amino acids 522-541 and 596-644 deleted from the Ti plasmid | This Study |
| EHA105*virE2::GFP11* | EHA105 derivative, with the GFP11-coding sequence inserted into *virE2* on pTiBo542 | (Li et al., 2014) |
| EHA105*virE2::GFP11*ΔVirE3(522-541) | EHA105*virE2::GFP11* derivative, with the sequences encoding the VirE3 amino acids 522-541 deleted from the Ti plasmid | This Study |
| EHA105*virE2::GFP11*ΔVirE3(598-644) | EHA105*virE2::GFP11* derivative, with the sequences encoding the VirE3 amino acids 598-644 deleted from the Ti plasmid | (Li et al., 2018) |
| EHA105*virE2::GFP11*ΔVirE3(522-541,598-644) | EHA105*virE2::GFP11* derivative, with the sequences encoding the VirE3 amino acids 522-541 and 598-644 deleted from the Ti plasmid | This Study |
| EHA105*virE2::GFP11*ΔVirE3 | EHA105*virE2::GFP11* derivative, with *virE3* deleted from the Ti plasmid | (Li et al., 2018) |
| EHA105*virE2::sfCherry11* | EHA105 derivative, with the sfCherry11-coding sequence inserted into *virE2* on pTiBo542 | This Study |
| EHA105*virE3::GFP11* | EHA105 derivative, with the GFP11-coding sequence inserted into *virE3* on pTiBo542 | (Li et al., 2018) |
| EHA105*virE3::GFP11*ΔVirE3(522-541) | EHA105*virE3::GFP11* derivative, with the sequences encoding the VirE3 amino acids 522-541 deleted from the Ti plasmid | This Study |
| EHA105*virE3::GFP11*ΔVirE3(598-644) | EHA105*virE3::GFP11* derivative, with the sequences encoding the VirE3 amino acids 598-644 deleted from the Ti plasmid | (Li et al., 2018) |
| EHA105*virE3::GFP11*ΔVirE3(522-541,598-644) | EHA105*virE3::GFP11* derivative, with the sequences encoding the VirE3 amino acids 522-541 and 598-644 deleted from the Ti plasmid | This Study |
| EHA105-VirE2::sfCherry11-VirE3::GFP11 | EHA105*virE3::GFP11* derivative, with the sfCherry11-coding sequence inserted into *virE2* on the Ti plasmid | This Study |

**Table S2.** Plasmids used in this study

| **Plasmids** | **Relevant characteristics** | **Source** |
| --- | --- | --- |
| pm-rb | A binary plasmid containing a plant plasma membrane marker; Km^r^ | (Nelson et al., 2007) |
| pmC13-Reverse | A binary plasmid, with the mCherry coding sequence placed downstream of the CaMV 35S promoter; Km^r^ | (Li et al., 2018) |
| pXY01 | A binary plasmid for target gene expression under the control of CaMV 35S promoter; Km^r^ | (Li and Pan, 2017) |
| pGFP1-10 | A binary plasmid, with the GFP1–10 coding sequence placed downstream of the CaMV 35S promoter; Km^r^ | (Li and Pan, 2017) |
| psfCherry1-10 | pXY01 derivative, with the sfCherry1–10 coding sequence placed downstream of the CaMV 35S promoter; Km^r^ | This Study |
| pQH308A | A binary plasmid, with the GFP1–10 and DsRed coding sequences on T-DNA; Km^r^ | (Li et al., 2014) |
| pQH308GR | pQH308A derivative, with the GFP1–10 and sfCherry1–10 coding sequences on T-DNA; Km^r^ | This Study |
| pGADT7 | Yeast two-hybrid cloning plasmid, with the GAL4 AD domain; Amp^r^ | Clontech |
| pGADT7-VirE3 | pGADT7 derivative, with the VirE3 coding sequence fused to the GAL4 AD domain; Amp^r^ | (Li et al., 2018) |
| pGADT7-VirE3(510-551) | pGADT7 derivative, with the VirE3(510-551) coding sequence fused to the GAL4 AD domain; Amp^r^ | This Study |
| pGADT7-VirE3(596-648) | pGADT7 derivative, with the VirE3(596-648) coding sequence fused to the GAL4 AD domain; Amp^r^ | This Study |
| pGADT7-VirE3(649-672) | pGADT7 derivative, with the VirE3(649-672) coding sequence fused to the GAL4 AD domain; Amp^r^ | This Study |
| pGBKT7 | Yeast two-hybrid cloning plasmid, with the GAL4 BD domain; Km^r^ | Clontech |
| pGBKT7-VirE2 | pGBKT7 derivative, with the VirE2 coding sequence fused to the GAL4 BD domain; Km^r^ | (Li et al., 2018) |
| pMAL-c2x | MBP tag expression vector; Amp^r^ | New England Biolabs |
| pMAL-VirE3(510-551) | pMAL-c2x derivative, with the VirE3(510-551) coding sequence fused to the MBP coding sequence; Amp^r^ | This Study |
| pMAL-VirE3(596-648) | pMAL-c2x derivative, with the VirE3(598-648) coding sequence fused to the MBP coding sequence; Amp^r^ | This Study |
| pRSET-A | Protein expression vector; Amp^r^ | Invitrogen |
| pRSET-E2 | pRSET-A derivative, with the VirE2 coding sequence at downstream of the T7 promoter; | This Study |
| pEx18Km | Counter-selectable plasmid carrying *sac*B marker; Km^r^ | (Li et al., 2014) |
| pEx18Km-ΔVirE3(522-541) | pEx18Km derivative, to delete the sequence encoding the VirE3 amino acids 522-541 on pTiBo542; Km^r^ | This Study |
| pEx18Km-ΔVirE3::S11(522-541) | pEx18Km derivative, to delete the sequence encoding the VirE3 amino acids 522-541 on the Ti plasmid; Km^r^ | This Study |
| pEx18Km-ΔVirE3(522-541,598-644) | pEx18Km derivative, to delete the sequence encoding the VirE3 amino acids 522-541 and 598-644 on pTiBo542; Km^r^ | This Study |
| pEx18Km-ΔVirE3::S11(522-541,598-644) | pEx18Km derivative, to delete the sequence encoding the VirE3 amino acids 522-541 and 598-644 on the Ti plasmid; Km^r^ | This Study |
| pEx18Km-VirE2::sfCherry11 | pEx18Km derivative, to insert the sfCherry11 coding sequence into the *virE2* on pTiBo542; Km^r^ | This Study |

**Table S3.** Primers used in this study

| **Primers** | **Sequences (5′ to 3′)** |
| --- | --- |
| E1001 | CGGGTTGCGGAGGATTTTC |
| E1002 | CTCGGCTCTCTCGTACTGCTCCACGATGGTGTAAGGGCTCCCTGATTGTACGTC |
| E1003 | GCAGTACGAGAGAGCCGAGGCCAGACACAGCACCACCCGAACGGAAGTTGTAAGC |
| E1004 | GCGCCTCTTCTTCGGTAATCA |
| E1005 | CTAGTCTAGAATGGCCATCATCAAGCCG |
| E1006 | CCGCTCGAGTCAAAAGCTGTTGACGCTTTG |
| E1007 | AGGCTCTTTCAACCTGTATCCG |
| E1008 | CCACAGAAACATTGTCCGAGGATGAAGACATCA |
| E1009 | CTCGGACAATGTTTCTGTGGCAATACATGATGATC |
| E1010 | GCAGGCCAGGGCTATACGAC |
| E1011 | CTAGTCTAGATTTTCCAAGATTTCAGTGAAGGC |
| E1012 | ACGCGTCGACTCGGATGGCCG |
| P1001 | GCTCTAGAATGGAGGAGGACAACATGGC |
| P1002 | CGCGGATCCTCAGTCCTCGTTGTGGCTGG |
| P1003 | CGCGGATCCTCTGCGAAGGACCGGTGATG |
| P1004 | CCGCTCGAGCTACTCTTGATCATCATGTATTGCCAC |
| P1005 | CGCGGATCCTCGGGTTTATTGACCCGCAATC |
| P1006 | CCGCTCGAGTTACGGTGAAGGAACCGGCAG |
| P1007 | CGCGGATCCTCAAGCCCGAATGCGCGA |
| P1008 | CCGCTCGAGTTAGAACCCTCTGGAGGTGGAA |
| P1009 | CGCGGATCCTGCGAAGGACCGGTGATG |
| P1010 | CGCGGATCCGGGTTTATTGACCCGCAATC |
| P1011 | CTAGTCTAGAAATAATTTTGTTTAACTTTAAGAAGGAGATATACAT ATGGATCCGTCTAGCAATGAG |
| P1012 | CGGGGTACCTCAAAAGCTGTTGACGCTTTG |

**References**

Hood, E.E., Gelvin, S.B., Melchers, L.S., and Hoekema, A. (1993). New*Agrobacterium* helper plasmids for gene transfer to plants. *Transgenic Research* 2(4)**,** 208-218.

Li, X., and Pan, S.Q. (2017). *Agrobacterium* delivers VirE2 protein into host cells via clathrin-mediated endocytosis. *Sci Adv* 3(3)**,** e1601528. doi: 10.1126/sciadv.1601528.

Li, X., Tu, H., and Pan, S.Q. (2018). *Agrobacterium* Delivers Anchorage Protein VirE3 for Companion VirE2 to Aggregate at Host Entry Sites for T-DNA Protection. *Cell Rep* 25(2)**,** 302-311 e306. doi: 10.1016/j.celrep.2018.09.023.

Li, X., Yang, Q., Tu, H., Lim, Z., and Pan, S.Q. (2014). Direct visualization of *Agrobacterium*-delivered VirE2 in recipient cells. *Plant J* 77(3)**,** 487-495. doi: 10.1111/tpj.12397.

Nelson, B.K., Cai, X., and Nebenfuhr, A. (2007). A multicolored set of *in vivo* organelle markers for co-localization studies in *Arabidopsis* and other plants. *Plant J* 51(6)**,** 1126-1136. doi: 10.1111/j.1365-313X.2007.03212.x.
